# Human Hemoglobin Subunit Beta Functions as a Pleiotropic Regulator of RIG-I/MDA5-Mediated Antiviral Innate Immune Responses

**DOI:** 10.1128/JVI.00718-19

**Published:** 2019-07-30

**Authors:** Qian Yang, Si-Yu Bai, Lian-Feng Li, Su Li, Yuexiu Zhang, Muhammad Munir, Hua-Ji Qiu

**Affiliations:** a State Key Laboratory of Veterinary Biotechnology, Harbin Veterinary Research Institute, Chinese Academy of Agricultural Sciences, Harbin, China; b Division of Biomedical and Life Sciences, Faculty of Health and Medicine, Lancaster University, United Kingdom; Hudson Institute of Medical Research

**Keywords:** MDA5-dsRNA interaction, RIG-I/MDA5-mediated signaling pathways, RNA viruses, pleiotropic regulator, human hemoglobin subunit beta, reactive oxygen species, ubiquitination

## Abstract

Hemoglobin is an important oxygen-carrying protein and plays crucial roles in establishing host resistance against pathogens and in regulating innate immune responses. The hemoglobin subunit beta (HB) is an essential component of hemoglobin, and we have previously demonstrated that the antiviral role of the porcine HB (pHB) is mediated by promoting type I interferon pathways. Thus, considering the high homology between human HB (hHB) and pHB, we hypothesized that hHB also plays an important role in the antiviral innate immunity. In this study, we characterized hHB as a regulatory factor for the replication of RNA viruses by differentially regulating the RIG-I- and MDA5-mediated antiviral signaling pathways. Furthermore, we showed that hHB directly inhibited MDA5-mediated signaling by reducing the MDA5–double-stranded RNA (dsRNA) interaction. Additionally, hHB required hHB-induced reactive oxygen species (ROS) to promote RIG-I-mediated signaling through enhancement of K63-linked RIG-I ubiquitination. Taken together, our findings suggest that hHB is a pleiotropic regulator of RIG-I/MDA5-mediated antiviral responses and further highlight the importance of the intercellular microenvironment, including the redox state, in regulating antiviral innate immune responses.

**IMPORTANCE** Hemoglobin, the most important oxygen-carrying protein, is involved in the regulation of innate immune responses. We have previously reported that the porcine hemoglobin subunit beta (HB) exerts antiviral activity through regulation of type I interferon production. However, the antiviral activities and the underlying mechanisms of HBs originating from other animals have been poorly understood. Here, we identified human HB (hHB) as a pleiotropic regulator of the replication of RNA viruses through regulation of RIG-I/MDA5-mediated signaling pathways. hHB enhances RIG-I-mediated antiviral responses by promoting RIG-I ubiquitination depending on the hHB-induced reactive oxygen species (ROS), while it blocks MDA5-mediated antiviral signaling by suppressing the MDA5-dsRNA interaction. Our results contribute to an understanding of the crucial roles of hHB in the regulation of the RIG-I/MDA5-mediated signaling pathways. We also provide novel insight into the correlation of the intercellular redox state with the regulation of antiviral innate immunity.

## Abstract (precis)

Hemoglobin, the most important oxygen-carrying protein, is involved in the regulation of innate immune responses. We have previously reported that the porcine hemoglobin subunit beta (HB) exerts antiviral activity through regulation of type I interferon production. However, the antiviral activities and the underlying mechanisms of HBs originating from other animals have been poorly understood. Here, we identified human HB (hHB) as a pleiotropic regulator of the replication of RNA viruses through regulation of RIG-I/MDA5-mediated signaling pathways. hHB enhances RIG-I-mediated antiviral responses by promoting RIG-I ubiquitination depending on the hHB-induced reactive oxygen species (ROS), while it blocks MDA5-mediated antiviral signaling by suppressing the MDA5-dsRNA interaction. Our results contribute to an understanding of the crucial roles of hHB in the regulation of the RIG-I/MDA5-mediated signaling pathways. We also provide novel insight into the correlation of the intercellular redox state with the regulation of antiviral innate immunity.

## INTRODUCTION

Type I interferons (IFNs) establish the first line of defense against viruses (1). They are activated in response to signaling cascades initiated by effective interactions between the genetically encoded host pattern recognition receptors (PRRs) and pathogen-associated molecular patterns (PAMPs) (2). Viral nucleic acid is one of the well-characterized PAMPs. Depending on the genetic nature of viruses, retinoic acid-inducible gene I (RIG-I)-like receptors (RLRs) function as the key viral RNA sensors and mediators of IFN production (3, 4).

RIG-I and melanoma differentiation-associated gene 5 (MDA5) are major members of RLRs and contain a central DEXD/H box helicase domain, which is responsible for recognizing viral RNA, and two caspase recruitment domains (CARD) at their N-terminal regions (5). Upon sensing viral RNA, RIG-I and MDA5 undergo conformational alterations and interact with the adaptor mitochondrial antiviral signaling protein (MAVS, also called IPS-1, VISA, or Cardif) through CARD (6, 7). Acting as a central adaptor, MAVS initiates downstream antiviral signaling by activating the downstream IKKα, -β, and -γ and TBK1/IKKi kinases, resulting in the activation of NF-κB and interferon regulatory factor 3 (IRF3)/IRF7 to transcriptionally induce type I IFNs (8–10).

RIG-I and MDA5 share high structural homologies and signaling features (11). However, they sense different species and types of viral RNAs (12). It is now well established that RIG-I primarily senses the 5′-triphosphate (5′PPP)-containing viral RNAs and some specific sequence motifs in the viral RNA, such as poly(U/UC) (13–15). In contrast to motifs recognized by RIG-I, the characteristics of viral PAMPs sensed by MDA5 remain elusive. It has been proposed that MDA5 can recognize long double-stranded RNA (dsRNA) as well as web-like RNA aggregates (16). As a countermeasure, it has been shown that RIG-I- and MDA5-mediated signaling pathways are differentially regulated (5). Some viral proteins exert different effects on the RIG-I- and MDA5-mediated pathways. For instance, the paramyxovirus V protein regulates the MDA5- but not RIG-I-mediated signaling (17). In addition to viral proteins, numerous host molecules have been identified to regulate the RIG-I-mediated signaling. The ARF-like protein 16 (Arl16) and the anti-apoptotic protein A20 interact with RIG-I to inhibit antiviral responses (18, 19); cylindromatosis (CYLD) and ubiquitin-specific peptidase 21 (USP21) remove K63-linked polyubiquitin chains to suppress RIG-I-mediated signaling (20, 21), and ring-finger protein 125 (RNF125) can trigger the proteasome-mediated degradation of RIG-I (22). Several MDA5-associated host proteins have also been identified, such as ADP-ribosylation factor-like protein 5B (Arl5B) and dihydroacetone kinase (DAK) (23, 24). However, the regulatory mechanisms of these two RLRs are insufficiently understood.

Hemoglobin is the main oxygen-carrying protein in vertebrates and many invertebrates, and in adult humans, it exists as a tetramer composed of two α-chains and two β-chains (25). It was previously thought that hemoglobin is expressed only by erythroid cells. However, this belief has been challenged by the recent findings that hemoglobin is expressed in a wide variety of nonerythrocytes, including hepatocytes, alveolar cells, neuronal/glial cells, and endometrial cells (26–29). Hemoglobin exerts multiple functions and plays important roles in resistance to the invasion of pathogens and in the regulation of innate immunity (30, 31). Peptides derived from hemoglobin have great potential as therapeutic drug candidates (32, 33). However, the antiviral activities and the underlying mechanisms of hemoglobin have been poorly explored. Previously, we corroborated for the first time that porcine hemoglobin subunit beta (pHB) is able to suppress the growth of classical swine fever virus (CSFV) through the regulation of RIG-I-mediated type I IFN responses (34). However, the roles of the HBs of other species in innate immunity have not yet been determined. Due to the significant amino acid homology (84.4%) between human HB (hHB) and pHB, we speculated that they have functional homologies in regulating antiviral innate immunity.

In the present study, we identified hHB as a pleiotropic regulator of innate antiviral immunity through regulation of RIG-I/MDA5-mediated signaling pathways. We investigated the molecular mechanisms underlying hHB-induced differential regulation of RIG-I- and MDA5-mediated type I IFN responses in humans. Our results illustrate the importance of hHB in regulating antiviral responses and provide novel insights into functional differences in RIG-I- and MDA5-mediated antiviral innate immunity.

## RESULTS

### hHB is involved in the defense responses against RNA viruses.

**FIG 1:**
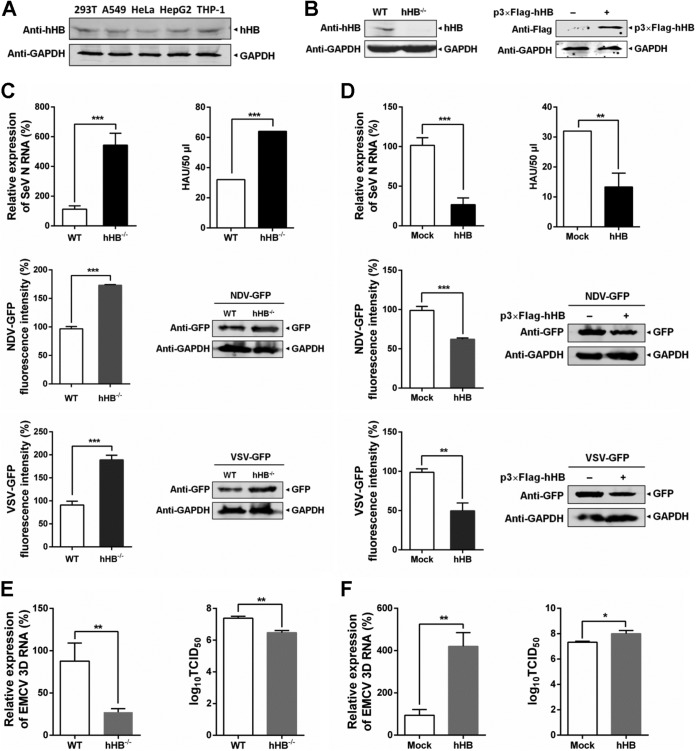
hHB is involved in the antiviral responses to RNA viruses. (A) The expression of hHB in different nonerythroid cell lines including HEK293T, HepG2, A549, HeLa, and THP-1. (B) The knockout efficiency of hHB in hHB-deficient HEK293T (hHB^−/−^) cells compared with the expression level of hHB in the wild-type HEK293T (WT). (C) hHB^−/−^ cells were more sensitive to Sendai virus (SeV), vesicular stomatitis virus (VSV), and Newcastle disease virus (NDV) infections. hHB^−/−^ and WT cells were infected with SeV for 24 h or with VSV-GFP or NDV-GFP for 48 h. Then the viral titers of SeV in the supernatants were determined by hemagglutination assay, and the RNA level of the SeV N protein in the cells was determined by real-time RT-PCR. In addition, the GFP expression and fluorescence levels in VSV-GFP- or NDV-GFP-infected cells were analyzed by Western blotting and an Enspire Multimode Plate Reader. HAU, hemagglutinating units. (D) Overexpression of hHB inhibited the replication of SeV, VSV, and NDV. HEK293T cells were transfected with the p3×Flag-CMV-10 empty vector (p3×Flag-EV) or p3×Flag-hHB for 24 h and then infected with SeV for 24 h or with VSV-GFP or NDV-GFP for 48 h. The viral titers of SeV in the supernatants and the RNA level of the SeV N protein in the cells or GFP expression and fluorescence of VSV-GFP or NDV-GFP were tested. (E) The effect of hHB on the replication of encephalomyocarditis virus (EMCV). hHB^−/−^ and WT cells were infected with EMCV for 24 h. Then viral titers (as 50% tissue culture infective dose [TCID_50_]) in the supernatants and the RNA level of the EMCV 3D protein in the cells were determined. (F) The effect of hHB overexpression on the replication of EMCV. HEK293T cells were infected with EMCV for 24 h. Then the titers in the supernatants and the RNA level of EMCV 3D protein in the cells were determined. The data represent the means ± standard deviations from three independent experiments. Significant differences are denoted as follows: *, *P* < 0.05; **, *P* < 0.01; ***, *P* < 0.001.

To verify whether hHB modulates host antiviral responses, we first evaluated the content of hHB in different nonerythroid cell lines which can simulate the expression of hHB in primary cells. hHB was observed in cell lines derived from various tissue, including kidney cells (HEK293T), liver cells (HepG2), lung cells (A549), cervix cells (HeLa), and peripheral blood mononuclear cells (THP-1) (Fig. 1A). Then, we used HEK293T cells as a model to investigate the hHB-mediated regulation of RIG-I/MDA5-mediated antiviral responses. To investigate the effect of hHB on infection by RNA viruses, we generated hHB-deficient HEK293T (hHB^−/−^) cells using the CRISPR/Cas9 system (Fig. 1B). Higher viral titers and RNA replication levels were observed in hHB^−/−^ cells when they were infected with Sendai virus (SeV), and the replication of vesicular stomatitis virus (VSV) or Newcastle disease virus (NDV) in hHB^−/−^ cells was higher than that in wild-type (WT) HEK293T cells (Fig. 1C). Accordingly, overexpression of hHB led to resistance to SeV and inhibited the replication of VSV and NDV (Fig. 1D). However, unexpectedly, the viral titers and level of RNA replication of encephalomyocarditis virus (EMCV) in hHB^−/−^ cells were lower than levels in WT cells, and the overexpression of hHB enhanced the growth of EMCV (Fig. 1E and F). Taken together, these results indicate that hHB can regulate the replication of RNA viruses.

### hHB promotes the RIG-I-mediated antiviral signaling.

**FIG 2:**
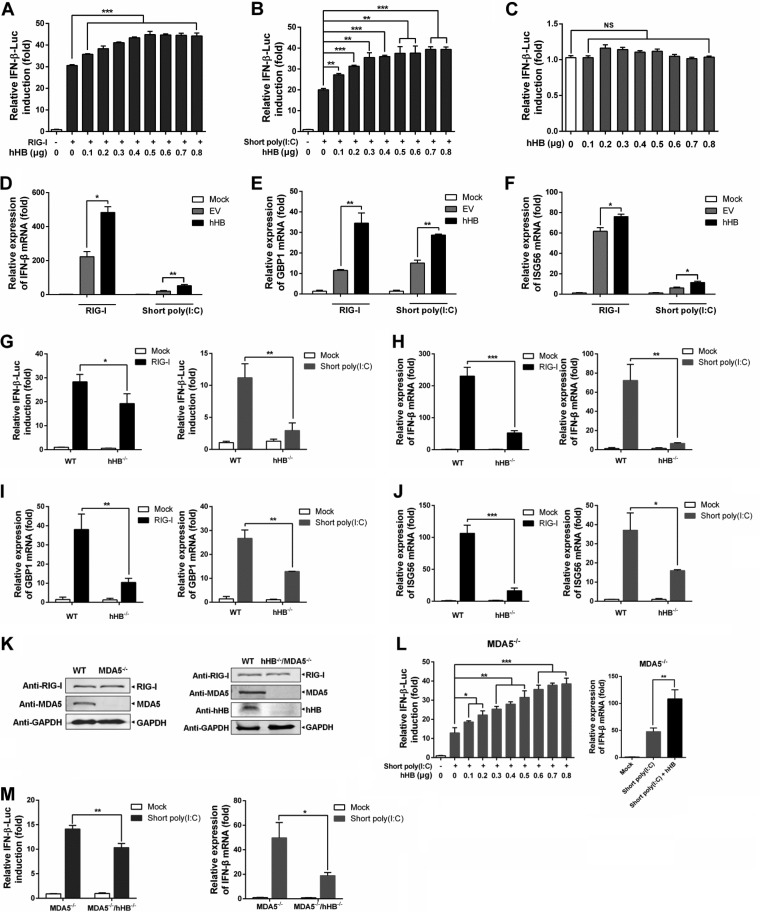
hHB enhances the RIG-I-mediated antiviral signaling. (A and B) Overexpression of hHB upregulated RIG-I-mediated activation of the IFN-β promoter. HEK293T cells were cotransfected with the indicated amounts of p3×Flag-hHB (hHB), pMyc-RIG-I (A) or short poly(I·C) (B), TK-*Renilla* luciferase (RLuc) internal reference reporter plasmid (pRLuc-TK), and IFN-β promoter firefly luciferase (FLuc) reporter plasmid (pIFN-β-FLuc) for 24 h. The activation of the IFN-β promoter was presented as luciferase reporter gene induction mediated by the IFN-β promoter and calculated as the relative levels of FLuc/RLuc. (C) The effect of hHB on the IFN-β promoter activation. HEK293T cells were cotransfected with the indicated amounts of p3×Flag-hHB, pRLuc-TK, and pIFN-β-FLuc for 24 h. The IFN-β promoter activation was tested. (D to F) Overexpression of hHB enhanced the transcription of IFN-β, GBP1, and ISG56. HEK293T cells were cotransfected with p3×Flag-EV (EV) or p3×Flag-hHB (hHB) and pMyc-RIG-I or short poly(I·C) for 24 h, and the IFN-β, GBP1, and ISG56 mRNA levels in cells were analyzed using real-time RT-PCR, as indicated. (G) The RIG-I-mediated activation of the IFN-β promoter in hHB^−/−^ cells. hHB^−/−^ and WT cells were cotransfected with pMyc-RIG-I or short poly(I·C) as well as pRLuc-TK and pIFN-β-FLuc. At 24 h posttransfection (hpt), the activation of IFN-β promoter was tested. (H to J) The RIG-I-mediated transcription of IFN-β, GBP1, and ISG56 in hHB^−/−^ cells. hHB^−/−^ and WT cells were transfected with pMyc-RIG-I or short poly(I·C). At 24 hpt, the IFN-β, GBP1, and ISG56 mRNA abundances, as indicated, were determined. (K) The knockout efficiency of MDA5 in WT and hHB^−/−^ cells. (L) Overexpression of hHB upregulated the short-poly(I·C)-induced transcription of IFN-β in MDA5-deficient HEK293T (MDA5^−/−^) cells. MDA5^−/−^ cells were cotransfected with the indicated amounts of p3×Flag-hHB, short poly(I·C), pRLuc-TK, and pIFN-β-FLuc for 24 h. The IFN-β promoter activation was tested. In addition, MDA5^−/−^ cells were cotransfected with p3×Flag-EV or p3×Flag-hHB and short poly(I·C) for 24 h, and the IFN-β mRNA levels were analyzed. (M) The effect of the deficiency of hHB on the short-poly(I·C)-mediated transcription of the IFN-β. Double-knockout cells deficient in MDA5 and hHB (MDA5^−/−^ hHB^−/−^) and MDA5^−/−^ cells were cotransfected with short poly(I·C) as well as pRLuc-TK and pIFN-β-FLuc for 24 h. Then the activation of IFN-β promoter was tested. In addition, MDA5^−/−^ hHB^−/−^ and MDA5^−/−^ cells were transfected with short poly(I·C). At 24 hpt, the IFN-β mRNA abundances were measured. The data represent the means ± standard deviations from three independent experiments. Significant differences are denoted as follows: *, *P* < 0.05; **, *P* < 0.01; ***, *P* < 0.001; NS, not significant (*P* > 0.05).

RIG-I and MDA5 function as key viral RNA sensors by recognizing different viral RNA species (12). While many negative-stranded RNA viruses, including SeV, VSV, and NDV, are mainly sensed by RIG-I, the viral RNA of picornaviruses such as EMCV is almost exclusively recognized by MDA5 (12). In conjunction with the aforesaid results, hHB exerted an antiviral function against SeV, VSV, and NDV. However, hHB appeared to be beneficial for the replication of EMCV. Therefore, we speculated that hHB may differentially regulate RIG-I- and MDA5-mediated antiviral immune responses. We first explored the contribution of hHB in RIG-I-mediated signaling. A dual-luciferase reporter system, including a thymidine kinase (TK)-*Renilla* luciferase (RLuc) internal reference reporter plasmid and an IFN-β promoter-firefly luciferase (FLuc) reporter plasmid, was used. The activation of the IFN-β promoter was presented as the level of luciferase reporter gene induction mediated by the IFN-β promoter and calculated as the relative level of FLuc/RLuc. We noticed that overexpression of hHB significantly enhanced RIG-I-induced activation of the IFN-β promoter, which was saturated at high concentrations (0.7 to 0.8 μg) (Fig. 2A). Many studies have shown that the length of dsRNA is important to differentially activate MDA5- or RIG-I-mediated antiviral signaling pathways in fibroblasts (35, 36). The poly(I·C) is a synthetic analog of dsRNA and is a molecular pattern associated with viral infection. Finally, low-molecular-weight poly(I·C) [known as short poly(I·C)] sensing required RIG-I whereas high-molecular-weight poly(I·C) [long poly(I·C)] sensing required MDA5 (37). Consistently, hHB also promoted IFN-β promoter activation in the cells stimulated with cytoplasmic short poly(I·C) (0.2 to 1 kb; an RIG-I agonist) (Fig. 2B). To exclude the possibility that hHB can activate IFN-β transcription by itself, we investigated the effect of hHB overexpression on the activation of the IFN-β promoter. The results showed that hHB was unable to activate the IFN-β promoter in HEK293T cells on its own (Fig. 2C). Moreover, overexpression of hHB upregulated RIG-I-induced or short-poly(I·C)-induced transcription of IFN-β and IFN-stimulated genes (ISGs) including GBP1 and ISG56 (Fig. 2D to F).

We also determined the impact of hHB deficiency on the RIG-I-mediated signaling pathway. Our results indicated that RIG-I-induced or short-poly(I·C)-induced activation of the IFN-β promoter was significantly impaired in hHB^−/−^ cells (Fig. 2G). Correspondingly, lower mRNA levels of IFN-β, GBP1, and ISG56 were observed in RIG-I-treated or short poly(I·C)-treated hHB^−/−^ cells (Fig. 2H to J). To further exclude the possible interference of the MDA5-mediated signaling pathway, we also generated MDA5-deficient HEK293T (MDA5^−/−^) and double-knockout cells deficient in MDA5 and hHB (MDA5^−/−^ hHB^−/−^) (Fig. 2K). Overexpression of hHB in MDA5^−/−^ cells promoted the short-poly(I·C)-induced activation of the IFN-β promoter in a dose-dependent manner and significantly enhanced the transcription of IFN-β mRNA (Fig. 2L). Moreover, the short-poly(I·C)-induced transcription of IFN-β was lower in MDA5^−/−^ hHB^−/−^ cells than that in the MDA5^−/−^ cells (Fig. 2M). These results highlight the involvement of RIG-I in hHB-mediated regulation of type I IFNs.

### hHB inhibits the MDA5-mediated antiviral signaling.

**FIG 3:**
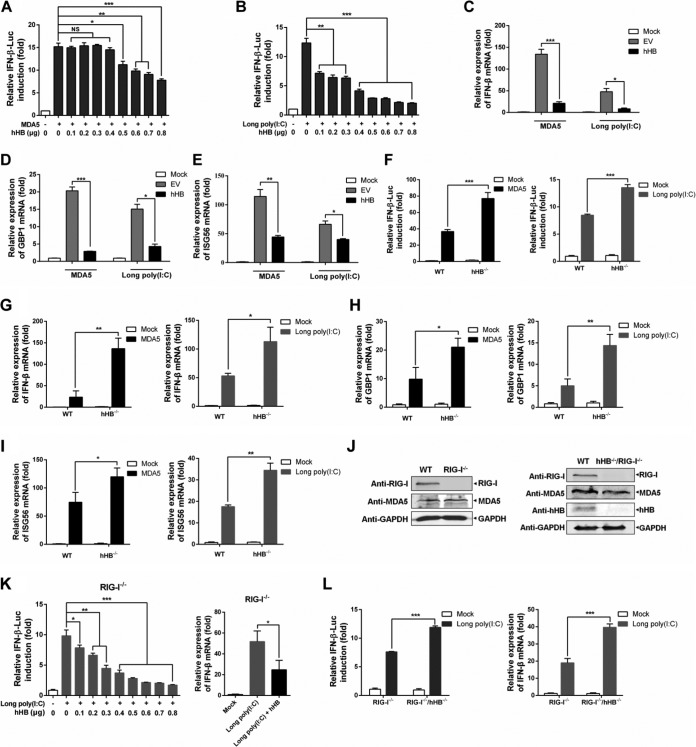
hHB inhibits the MDA5-mediated antiviral signaling. (A and B) Overexpression of hHB suppressed MDA5-mediated activation of the IFN-β promoter. HEK293T cells were cotransfected with the indicated amounts of p3×Flag-hHB, pMyc-MDA5 (A) or long poly(I·C) (B), pRLuc-TK, and pIFN-β-FLuc for 24 h. The activation of IFN-β promoter was examined. (C to E) Overexpression of hHB decreased the transcription of IFN-β, GBP1, and ISG56. HEK293T cells were transfected with the p3×Flag-EV or p3×Flag-hHB and pMyc-MDA5 or long poly(I·C) for 24 h, and the IFN-β, GBP1, and ISG56 mRNA levels in cells were analyzed, as indicated. (F) The MDA5-mediated activation of the IFN-β promoter in hHB^−/−^ cells. hHB^−/−^ and WT cells were cotransfected with pMyc-MDA5 or long poly(I·C) as well as pRLuc-TK and pIFN-β-FLuc. At 24 hpt, the activation of IFN-β promoter was analyzed. (G to I) The MDA5-mediated transcription of IFN-β, GBP1, and ISG56 in hHB^−/−^ cells. hHB^−/−^ and WT cells were transfected with pMyc-MDA5 or long poly(I·C) for 24 h. Then the IFN-β, GBP1, and ISG56 mRNA abundances, as indicated, were determined. (J) The knockout efficiency of RIG-I in WT and hHB^−/−^ cells. (K) Overexpression of hHB inhibited long-poly(I·C)-induced transcription of IFN-β in RIG-I-deficient HEK293T (RIG-I^−/−^) cells. RIG-I^−/−^ cells were cotransfected with the indicated amounts of p3×Flag-hHB, long poly(I·C), pRLuc-TK, and pIFN-β-FLuc for 24 h. The IFN-β promoter activation was then assessed. Additionally, RIG-I^−/−^ cells were cotransfected with p3×Flag-EV or p3×Flag-hHB and long poly(I·C) for 24 h, and the IFN-β mRNA levels were analyzed. (M) The effect of the deficiency of hHB on long-poly(I·C)-mediated transcription of the IFN-β. Double-knockout cells deficient in RIG-I and hHB (RIG-I^−/−^ hHB^−/−^) and RIG-I^−/−^ cells were cotransfected with long poly(I·C), pRLuc-TK, and pIFN-β-FLuc. At 24 hpt, the activation of IFN-β promoter was analyzed. In addition, RIG-I^−/−^ hHB^−/−^ and RIG-I^−/−^ cells were transfected with long poly(I·C) for 24 h. The IFN-β mRNA abundance was determined. The data represent the means ± standard deviations from three independent experiments. Significant differences are denoted as follows: *, *P* < 0.05; **, *P* < 0.01; ***, *P* < 0.001; NS, not significant (*P* > 0.05).

We next investigated the contribution of hHB in MDA5-mediated type I IFN signaling. We examined the activation of IFN-β promoter in p3×Flag-hHB and pMyc-MDA5 cotransfected HEK293T cells. The results demonstrated that overexpression of hHB significantly decreased MDA5-induced IFN-β promoter activation in a dose-dependent manner (Fig. 3A). The suppressive effects of hHB on IFN-β promoter activation were also evident in the cells stimulated with long poly(I·C) (1.5 to 8 kb; an MDA5 agonist) (Fig. 3B). Overexpression of hHB also inhibited the transcription of IFN-β, GBP1, and ISG56 induced by MDA5 or long poly(I·C) (Fig. 3C to E).

To further verify the contribution of hHB in MDA5-mediated signaling, we examined the impact of hHB knockout on IFN-β transcription. We observed that the activation of IFN-β promoter and the transcription of IFN-β, GBP1, and ISG56 in response to long poly(I·C) or MDA5 stimulation were significantly higher in the hHB^−/−^ cells than in the WT cells (Fig. 3F to I). We also generated RIG-I-deficient HEK293T (RIG-I^−/−^) cells and double-knockout cells deficient in both RIG-I and hHB (RIG-I^−/−^ hHB^−/−^) to exclude the possible disturbance of RIG-I (Fig. 3J). Overexpression of hHB in RIG-I^−/−^ cells inhibited long-poly(I·C)-induced activation of the IFN-β promoter in a dose-dependent manner and significantly suppressed the transcription of IFN-β mRNA (Fig. 3K). Moreover, long-poly(I·C)-induced transcription of IFN-β was higher in RIG-I^−/−^ hHB^−/−^ cells than that in the RIG-I^−/−^ cells (Fig. 3L). Based on these findings, it is plausible that hHB can distinctly regulate IFN-β production through MDA5- and RIG-I-mediated signaling pathways.

### hHB regulates the replication of RNA viruses through RIG-I- and MDA5-mediated signaling pathways.

**FIG 4:**
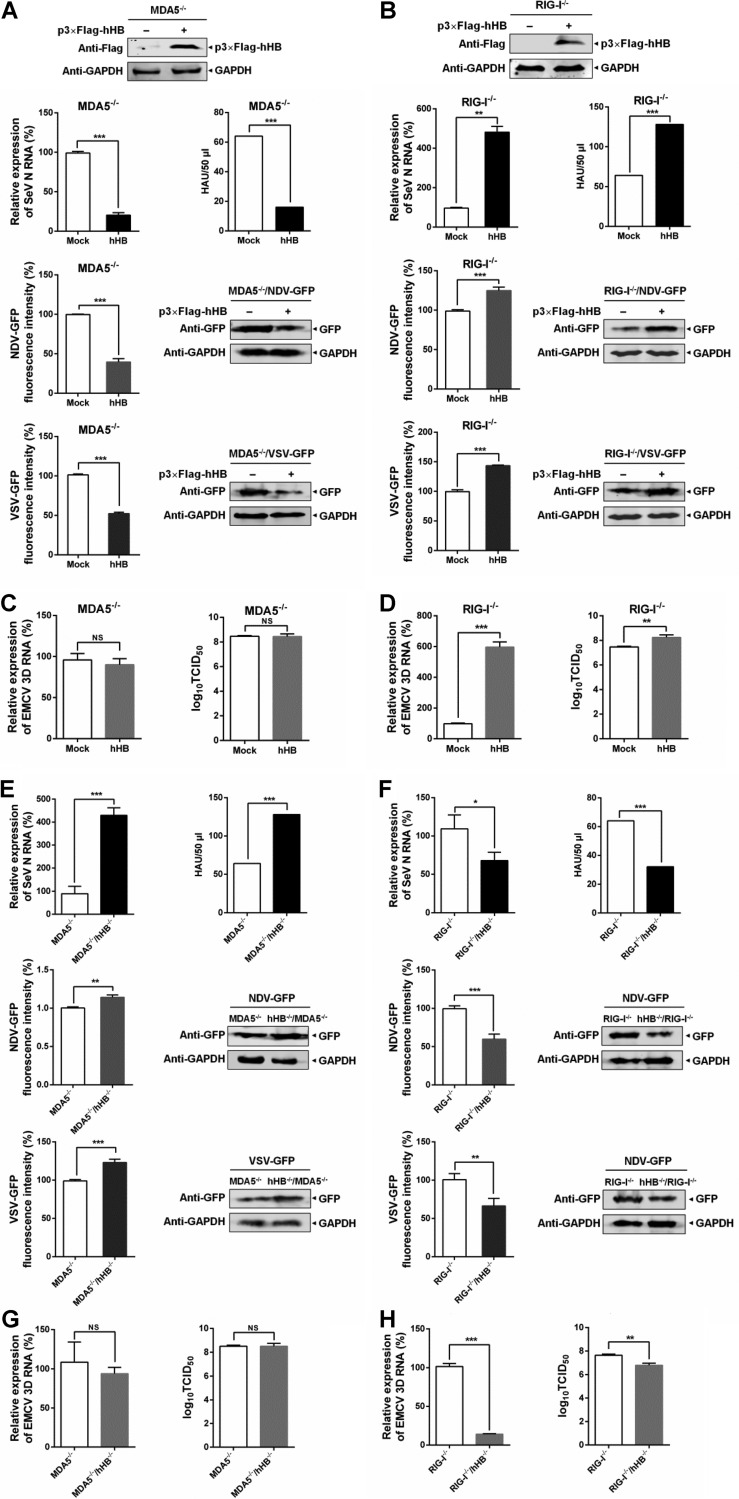
hHB regulates the RIG-I- and MDA5-mediated antiviral response to RNA viruses. (A and B) The effects of hHB overexpression on the replication of SeV, VSV, and NDV in MDA5^−/−^ and RIG-I^−/−^ cells. MDA5^−/−^ or RIG-I^−/−^ cells were transfected with p3×Flag-hHB for 24 h and then were infected with SeV for 24 h or with VSV-GFP or NDV-GFP for 48 h. The viral titers of SeV in the supernatants, the RNA level of the SeV N protein in cells, or GFP expression and fluorescence of VSV-GFP or NDV-GFP were determined. (C and D) The effect of hHB-overexpression on the replication of EMCV in MDA5^−/−^ and RIG-I^−/−^ cells. MDA5^−/−^ or RIG-I^−/−^ cells were infected with EMCV for 24 h. Then the titers in the supernatants and the RNA level of the EMCV 3D protein in the cells were determined. (E and F) Deficiency of hHB differently affected the replication of SeV, VSV, and NDV in RIG-I^−/−^ and MDA5^−/−^ cells. MDA5^−/−^ hHB^−/−^ cells and MDA5^−/−^ or RIG-I^−/−^ hHB^−/−^ and RIG-I^−/−^ cells, as indicated, were infected with SeV for 24 h or with VSV-GFP or NDV-GFP for 48 h. Then the viral titers of SeV in the supernatants, the RNA level of the SeV N protein in the cells, or the GFP expression and fluorescence of VSV-GFP or NDV-GFP were analyzed. (G and H) Deficiency of hHB differently affected the replication of EMCV in RIG-I^−/−^ and MDA5^−/−^ cells. MDA5^−/−^ hHB^−/−^ cells and MDA5^−/−^ cells or RIG-I^−/−^ hHB^−/−^ and RIG-I^−/−^ cells, as indicated, were infected with EMCV for 24 h. The titers in the supernatants and the RNA level of the EMCV 3D protein in the cells were determined. The data represent the means ± standard deviations from three independent experiments. Significant differences are denoted as follows: *, *P* < 0.05; **, *P* < 0.01; ***, *P* < 0.001; NS, not significant (*P* > 0.05).

We further verified the contribution of hHB-mediated regulation of the RIG-I- and MDA5-mediated signaling to the replication of RNA viruses. We first investigated the relevance of hHB overexpression and the replication of RNA viruses in MDA5^−/−^ and RIG-I^−/−^ cells. The results showed that overexpression of hHB led to resistance against SeV reproduction and inhibited the replication of SeV, VSV, and NDV in MDA5^−/−^ cells (Fig. 4A). Conversely, in RIG-I^−/−^ cells, overexpression of hHB enhanced the replication of SeV, VSV, and NDV (Fig. 4B). Moreover, although hHB overexpression also increased the viral titer and RNA replication of EMCV in RIG-I^−/−^ cells, it had little impact on the viral titer and RNA replication of EMCV in MDA5^−/−^ cells (Fig. 4C and D).

In order to further assess the combinatorial impacts of RLR and hHB on viruses, we compared the replication levels of SeV, VSV, NDV, and EMCV between MDA5^−/−^ hHB^−/−^ and MDA5^−/−^ cells and between RIG-I^−/−^ hHB^−/−^ and RIG-I^−/−^ cells. The MDA5^−/−^ hHB^−/−^ cells showed higher viral titers and RNA replication levels of SeV than MDA5^−/−^ cells, and the replication levels of VSV and NDV were also higher in MDA5^−/−^ hHB^−/−^ cells than levels in MDA5^−/−^ cells (Fig. 4E). In contrast, deficiency of hHB in RIG-I^−/−^ cells established resistance to SeV, VSV, and NDV (Fig. 4F). Additionally, deficiency of hHB suppressed the viral titer and RNA replication of EMCV in RIG-I^−/−^ cells but exhibited little effect on the replication of EMCV in MDA5^−/−^ cells (Fig. 4G and H). Taken together, these results indicate that hHB can differentially regulate the defense response of host cells to RNA viruses through the RIG-I- and MDA5-mediated antiviral signaling pathway.

### hHB has no effect on the expression of RIG-I or MDA5.

**FIG 5:**
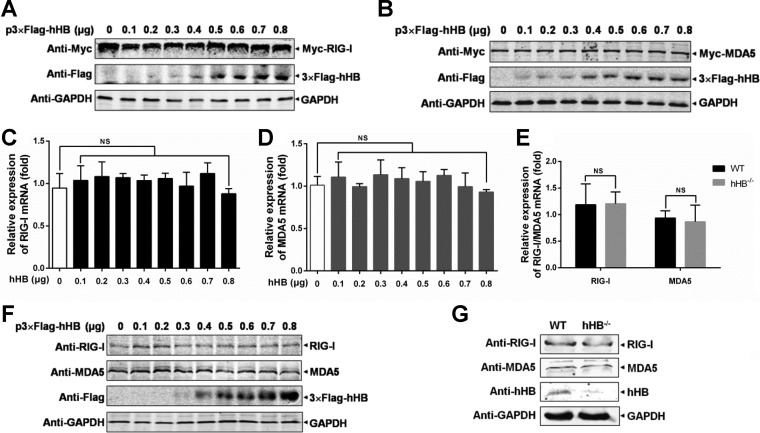
hHB does not affect RIG-I or MDA5 expression. (A and B) Overexpression of hHB had no effects on the exogenous protein expression of RIG-I or MDA5. HEK293T cells were cotransfected with the indicated amounts of p3×Flag-hHB and pMyc-RIG-I or pMyc-MDA5 for 24 h, and RIG-I and MDA5 expression was analyzed by Western blotting, as indicated. (C and D) Overexpression of hHB did not affect RIG-I and MDA5 transcription. HEK293T cells were transfected with the indicated amounts of p3×Flag-hHB (hHB) for 24 h, and then RIG-I and MDA5 mRNA levels in cells were analyzed as indicated using real-time RT-PCR. (E) The mRNA levels of RIG-I and MDA5 exhibited no difference between the hHB^−/−^ and WT cells. The mRNA levels of RIG-I and MDA5 in the cells were analyzed using real-time RT-PCR. (F) The endogenous protein expression of RIG-I and MDA5 was unchanged in hHB overexpression cells. HEK293T cells were transfected with the indicated amounts of p3×Flag-hHB for 48 h, and the RIG-I and MDA5 expression levels were analyzed by Western blotting. (G) The endogenous protein expression of RIG-I and MDA5 in the hHB^−/−^ and WT cells was examined by Western blotting. NS, not significant (*P* > 0.05).

After establishing the relationship between hHB and the MDA5/RIG-I pathways in regulating IFN-β, we explored whether hHB affects the expression of RIG-I or MDA5. Overexpression of hHB failed to alter the exogenous protein expression of RIG-I and MDA5 at various doses (Fig. 5A and B). In addition, the mRNA levels of RIG-I and MDA5 remained unchanged upon overexpression of hHB (Fig. 5C and D). We were unable to observe a difference between the mRNA levels of RIG-I or MDA5 in hHB^−/−^ and WT cells (Fig. 5E). Correspondingly, the endogenous expression of RIG-I and MDA5 remained unaffected in hHB-overexpressing cells (Fig. 5F). Moreover, there were no differences observed in the endogenous expression of RIG-I or MDA5 in hHB^−/−^ cells and the WT cells (Fig. 5G).

### hHB interferes with the MDA5-dsRNA interaction and enhances the ubiquitination of RIG-I.

**FIG 6:**
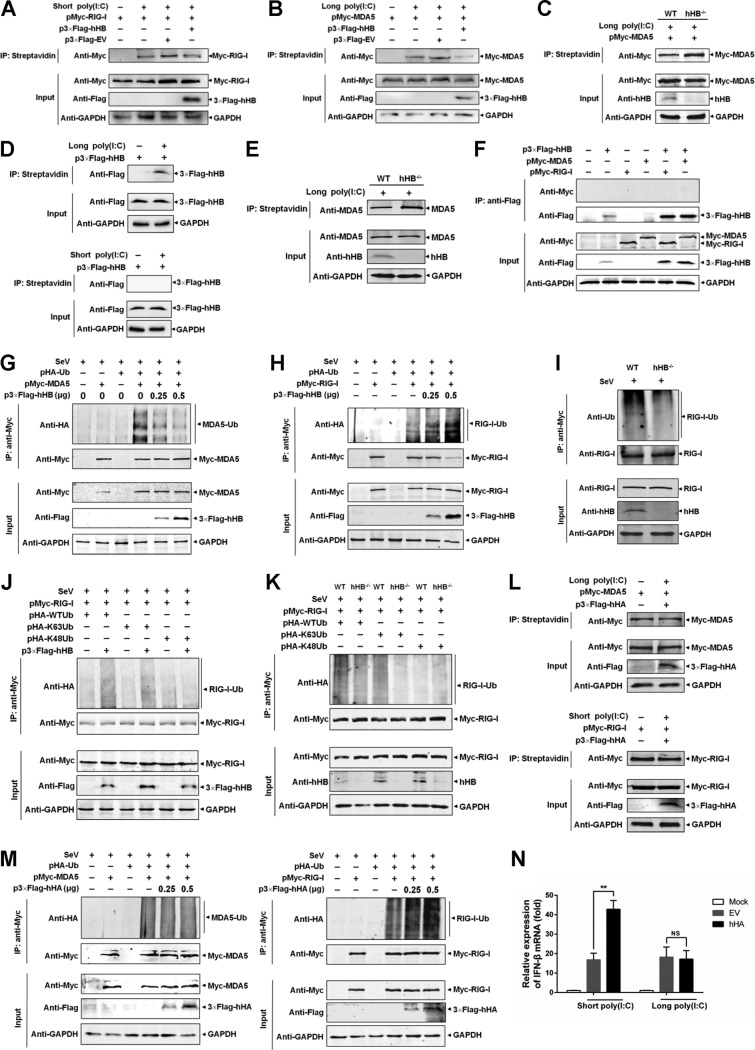
hHB inhibits the binding of dsRNA to MDA5 but not to RIG-I and enhances the ubiquitination of RIG-I. (A) hHB has no influence on the interaction of exogenous RIG-I with short poly(I·C). HEK293T cells were cotransfected with pMyc-RIG-I and p3×Flag-hHB for 48 h. Then the cells were lysed, and the cell lysates were incubated with the photobiotin-labeled short poly(I·C) for 4 h at 4°C. Then the biotinylated RNA-protein compounds were immunoprecipitated (IP) with Dynabeads Streptavidin for 1 h at room temperature. The bound Myc–RIG-I proteins were applied to SDS-PAGE gels and then analyzed by Western blotting. (B and C) hHB inhibits the binding of exogenous MDA5 to the long poly(I·C). HEK293T cells were transfected with pMyc-MDA5 and p3×Flag-hHB (B), or the hHB^−/−^ and the WT cells were transfected with pMyc-MDA5 (C). The cell lysates were collected at 48 hpt and incubated with the photobiotin-labeled long poly(I·C) for 4 h at 4°C. Then the RNA-bound proteins were precipitated and analyzed. (D) hHB inhibits the binding of endogenous MDA5 to the long poly(I·C). The lysates of the hHB^−/−^ and the WT cells were incubated with the photobiotin-labeled long poly(I·C) as described above. Then the long-poly(I·C)-bound proteins were precipitated and analyzed. (E) hHB interacted with long poly(I·C) but not short poly(I·C). HEK293T cells were transfected with p3×Flag-hHB. At 48 hpt, the cell lysates were collected, and the long-poly(I·C)-bound proteins were precipitated and analyzed as described above. (F) Co-IP analysis of interaction between hHB and RIG-I or MDA5 as described in Materials and Methods. (G and H) The effects of hHB on the ubiquitination of exogenous RIG-I and MDA5. HEK293T cells were cotransfected with the indicated plasmids for 12 h and then infected with SeV for 12 h. The prepared cell extracts were analyzed by IP analysis using an anti-Myc monoclonal antibody. (I) The endogenous ubiquitination of RIG-I in hHB^−/−^ cells. The hHB^−/−^ and WT cells were infected with SeV for 12 h. The prepared cell extracts were analyzed by IP analysis using an anti-RIG-I monoclonal antibody. (J and K) The effects of hHB on the K63-linked ubiquitination of RIG-I and MDA5. HEK293T cells or hHB^−/−^ cells were cotransfected with the indicated plasmids for 12 h and then infected with SeV for 12 h. The prepared cell extracts were analyzed by IP analysis using an anti-Myc monoclonal antibody. (L) hHA has no influence on the interactions of RIG-I–short poly(I·C) and MDA5–long poly(I·C). HEK293T cells were cotransfected with pMyc-RIG-I or pMyc-MDA5 and p3×Flag-hHA for 48 h. Then the cells were lysed, and the short-poly(I·C)- or long-poly(I·C)-bound proteins were precipitated and analyzed as described above. (M) The effect of hHA on the ubiquitination of RIG-I and MDA5. HEK293T cells were cotransfected with the indicated plasmids for 12 h and then infected with SeV for 12 h. The prepared cell extracts were analyzed by IP analysis using an anti-Myc monoclonal antibody. (N) Overexpression of hHA promoted the RIG-I-mediated activation of IFN-β transcription but not the MDA5-mediated activation of IFN-β transcription. HEK293T cells were transfected with p3×Flag-EV or p3×Flag-hHA and short poly(I·C) or long poly(I·C) for 24 h, and the IFN-β mRNA level in cells was analyzed. The data represent the means ± standard deviations from three independent experiments. Significant differences are denoted as follows: **, *P* < 0.01; NS, not significant (*P* > 0.05). Ub, ubiquitin; HA, hemagglutinin.

The results presented so far clearly articulate the involvement of RIG-I/MDA5 in mediating hHB-dependent regulation of the type I IFN pathway without affecting RIG-I/MDA5 protein expression. We next mechanistically investigated whether hHB affects the activation of these RLRs. Previous studies have demonstrated that sensing different types of viral RNA is required for the activation of MDA5 and RIG-I to initiate their signal transductions (38–40). RIG-I and MDA5 also recognize short poly(I·C) and long poly(I·C) as synthetic dsRNA analogues, respectively (35). Thus, we examined whether hHB affected the interaction between RIG-I or MDA5 and dsRNA using a short- or long-poly(I·C)-binding assay. We overexpressed exogenous hHB and RIG-I in HEK293T cells and examined the binding of the photobiotin-labeled short poly(I·C) with the exogenous RIG-I. The results showed that short poly(I·C) interacted with the exogenous RIG-I, which is independent of hHB (Fig. 6A). However, overexpression of hHB significantly inhibited the interaction of exogenous MDA5 with long poly(I·C) (Fig. 6B). Furthermore, exogenous MDA5 was also overexpressed in hHB^−/−^ and WT cells, and a poly(I·C)-binding assay indicated that long poly(I·C) interacted with exogenous MDA5 more effectively in the hHB^−/−^ cells than in the WT cells (Fig. 6C). These results indicate that hHB acts as a repressor of MDA5 activation by inhibiting the MDA5-dsRNA interaction. To further verify this possibility, we also used long poly(I·C) to pull down endogenous MDA5 in hHB^−/−^ and WT cells. As expected, the binding of long poly(I·C) with the endogenous MDA5 was also stronger in hHB^−/−^ cells than that in WT cells (Fig. 6D). Interestingly, when we determined the interaction between hHB and dsRNA, we found that hHB was precipitated with long poly(I·C) but not with short poly(I·C) (Fig. 6E). Due to the association of hHB with dsRNA and the involvement of hHB in MDA5-dsRNA interaction, we were interested to investigate if the interaction with the dsRNA occurs at the interface of hHB and MDA5. Coimmunoprecipitation (co-IP) analysis indicated no identifiable interaction of hHB with either MDA5 or RIG-I (Fig. 6F). These results underline the possibility that the binding of hHB to dsRNA may compete for the interaction of MDA5 with the dsRNA ligand and that this competition may result in a reduction of IFN-β induction.

Upon interaction with dsRNA ligands, RIG-I or MDA5 is ubiquitinated before recruitment to the mitochondrion-associated membrane and binding to MAVS (41). This suggested that the ubiquitination of RIG-I and MDA5 is crucial for the activation of RLR signaling. Thus, we overexpressed exogenous hHB, ubiquitin, and RIG-I or MDA5 in HEK293T cells to evaluate the ubiquitination of exogenous RIG-I and MDA5 upon hHB overexpression. Based on the disruption of the MDA5-dsRNA interaction by hHB, the ubiquitination of MDA5 was certainly suppressed by hHB in a dose-dependent manner (Fig. 6G). However, the ubiquitination of exogenous RIG-I was potentiated by increasing hHB protein expression (Fig. 6H). To further confirm the effect of hHB on the ubiquitination of RIG-I, we compared the levels of endogenous ubiquitination of RIG-I in hHB^−/−^ and WT cells after SeV infection. The results showed that the level of endogenous ubiquitination of RIG-I was lower in the hHB^−/−^ cells than that in the WT cells (Fig. 6I). It has been shown that RIG-I has different ubiquitination forms and that K63-linked ubiquitination of RIG-I is positively required for RIG-I activation, whereas K48-linked ubiquitination will result in the destabilization of RIG-I (22, 42). To verify if hHB promotes RIG-I activation through potentiating RIG-I ubiquitination, we constructed two ubiquitin mutants in which all lysine residues were replaced with arginine except K48 or K63 (HA-K48Ub or HA-K63Ub, respectively; HA is hemagglutinin and Ub is ubiquitin) to further investigate the polyubiquitination of RIG-I regulated by hHB. The results showed that hHB enhanced K63-linked but not K48-linked RIG-I ubiquitination (Fig. 6J). Moreover, the total ubiquitination of RIG-I and the K63-linked RIG-I ubiquitination were lower in hHB^−/−^ cells than levels in WT cells (Fig. 6K). In addition, to uncover whether the action of hHB is characteristic or noncharacteristic, we also examined whether the alpha subunit of hemoglobin (hHA) has similar effects on the activation of RIG-I or MDA5. The results showed that both the RIG-I–short poly(I·C) and the MDA5-long poly(I·C) interactions were independent of hHA (Fig. 6L). Furthermore, the ubiquitination of MDA5 remained unchanged by hHA, but the ubiquitination of RIG-I was enhanced by hHA in a dose-dependent manner (Fig. 6M). Moreover, hHA could promote the short-poly(I·C)-induced transcription of IFN-β but not the long-poly(I·C)-induced transcription of IFN-β (Fig. 6N). These data imply that the inhibition of the MDA5-dsRNA interaction is a characteristic of hHB.

### ROS is required for the hHB-induced upregulation of the RIG-I signaling pathway.

**FIG 7:**
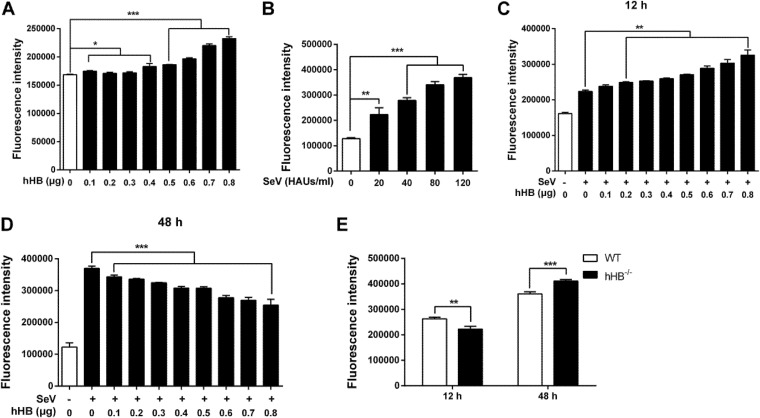
hHB and viral infections promote the intracellular ROS accumulation. (A) hHB increased intracellular ROS accumulation. HEK293T cells were transfected with p3×Flag-hHB at the indicated concentrations for 24 h, and then the cytoplasmic ROS formation was monitored by using the oxidant-sensitive fluorescent probe DCFH-DA as described in Materials and Methods. (B) SeV infection promoted intracellular ROS accumulation. HEK293T cells were infected with 10, 20, 30, 40, or 50 hemagglutinin units (HAU)/ml SeV for 24 h. Then the cytoplasmic ROS formation was determined. (C and D) The effect of hHB on ROS accumulation in SeV-infected cells. HEK293T cells were transfected with p3×Flag-hHB at the indicated concentrations for 24 h and then treated with 20 HAUs/ml SeV. Then the cytoplasmic ROS formation was measured at 12 hand 48 h after infection. (E) The SeV-induced ROS accumulation in hHB^−/−^ cells. WT cells and hHB^−/−^ cells were infected with SeV, and the cytoplasmic ROS formation was measured at 12 h and 48 h after infection. The data represent the means ± standard deviations from three independent experiments. Significant differences are denoted as follows: *, *P* < 0.05; **, *P* < 0.01; ***, *P* < 0.001.

Considering that hemoglobin is able to regulate the production of reactive oxygen species (ROS), which is a key factor for the host cell to trigger efficient activation of immunity (43–45), we evaluated if ROS was involved in the hHB-mediated regulation of the RIG-I or MDA5 signaling pathway. So, we first explored the links among hHB, ROS, and virus infections. hHB was overexpressed in HEK293T cells, and ROS production was monitored using the oxidant-sensitive fluorescent detection probe dichlorodihydrofluorescein diacetate (DCFH-DA). The results demonstrated that hHB increased intracellular ROS accumulation in a dose-dependent manner (Fig. 7A). In addition, intracellular ROS accumulation was also upregulated by SeV in a dose-dependent manner (Fig. 7B). Thus, we also monitored the effect of hHB on ROS accumulation in SeV-infected cells. The overexpression of hHB promoted ROS production in a dose-dependent manner at an early time of SeV infection (Fig. 7C). However, intracellular ROS accumulation was far more impacted by robust viral replication than by hHB at a later stage of SeV infection. Thus, the hHB-overexpressed cells showed lower intracellular ROS accumulation at a later time of SeV infection as the replication of SeV was suppressed (Fig. 7D). Consistently, SeV induced lower ROS production in hHB^−/−^ cells than in WT cells at the earlier time, but the hHB^−/−^ cells accumulated more intracellular ROS at the later time of SeV infection (Fig. 7E).

**FIG 8:**
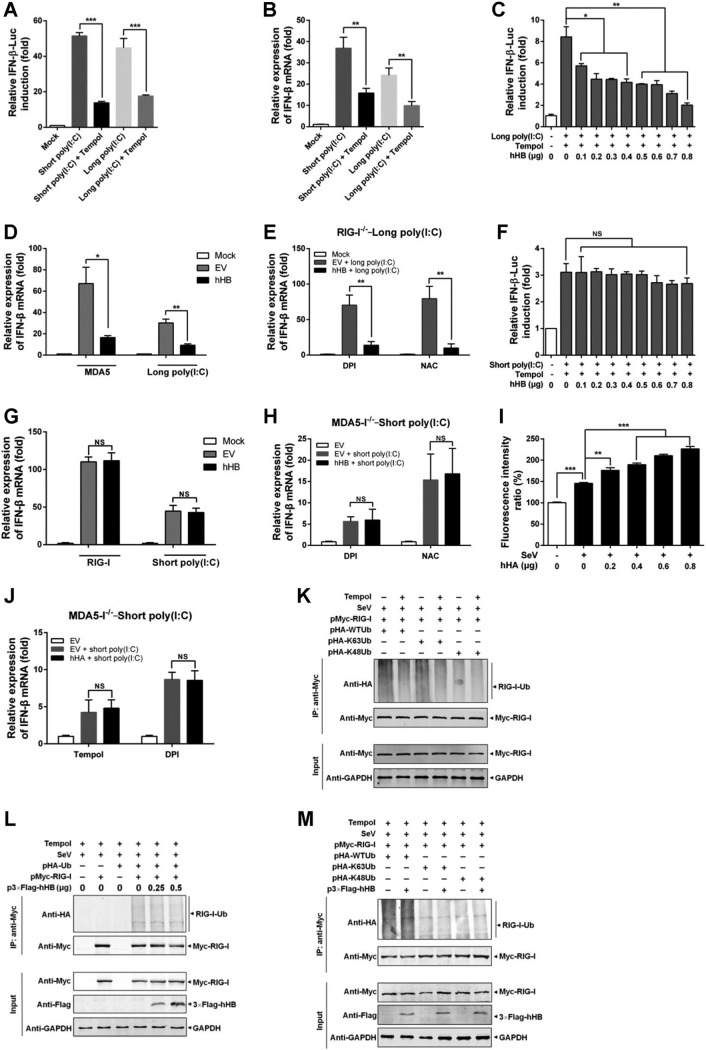
Tempol inhibits hHB-induced facilitation of the RIG-I signaling pathway but has no effect on hHB-induced inhibition of the MDA5 signaling pathway. (A) The effects of tempol on long-poly(I·C)- or short-poly(I·C)-induced activation of the IFN-β promoter. HEK293T cells were transfected with pRLuc-TK, pIFN-β-FLuc, and long poly(I·C) or short poly(I·C) for 12 h and then treated with 3 mM tempol or PBS for 12 h. The activation of IFN-β promoter was tested. (B) The effects of tempol on long-poly(I·C)- or short-poly(I·C)-induced IFN-β mRNA transcription. HEK293T cells were transfected with long poly(I·C) or short poly(I·C) for 12 h and then treated with 3 mM tempol or PBS for 12 h. The IFN-β mRNA level in cells was analyzed. (C) Tempol suppressed hHB-induced inhibition of the activation of the IFN-β promoter in response to long poly(I·C). HEK293T cells were transfected with the 3×Flag-hHB (hHB) at the indicated concentrations, pRLuc-TK, pIFN-β-FLuc, and long poly(I·C) for 12 h. Then the cells were treated with 3 mM tempol or PBS for 24 h. (D) The effect of tempol on hHB regulation of MDA5-mediated transcription of IFN-β. HEK293T cells were transfected with the p3×Flag-EV or p3×Flag-hHB and pMyc-MDA5 or long poly(I·C) for 12 h. Then the cells were treated with 3 mM tempol or PBS for 12 h. (E) The effect of diphenyleneiodonium chloride (DPI) and *N*-acetyl-l-cysteine (NAC) on the hHB regulation of MDA5-mediated mRNA transcription of IFN-β. RIG-I^−/−^ cells were transfected with the p3×Flag-EV or p3×Flag-hHB and/or long poly(I·C) for 12 h. Then the cells were treated with 3 μM DPI, 10 mM NAC, or PBS for 12 h. (F) Tempol suppressed hHB-induced upregulation of the activation of the IFN-β promoter in response to short poly(I·C). HEK293T cells were transfected with p3×Flag-hHB (hHB) at the indicated concentrations, pRLuc-TK, pIFN-β-FLuc, and short poly(I·C) for 12 h. Then cells were treated with 3 mM tempol or PBS for 24 h. (G) The effect of tempol on the regulation of RIG-I-mediated IFN-β transcription by hHB. HEK293T cells were transfected with the p3×Flag-EV or p3×Flag-hHB and pMyc-RIG-I or short poly(I·C) for 12 h. Then the cells were treated with 3 mM tempol or PBS for 12 h. (H) The effect of DPI and NAC on the hHB regulation of RIG-I-mediated mRNA transcription of IFN-β. MDA5^−/−^ cells were transfected with the p3×Flag-EV or p3×Flag-hHB and short poly(I·C) for 12 h. Then the cells were treated with 3 μM DPI, 10 mM NAC, or PBS for 12 h. (I) The effect of hHA on the ROS accumulation in SeV-infected cells. HEK293T cells were transfected with p3×Flag-hHA at the indicated concentrations for 24 h and then were infected with SeV. Then the cytoplasmic ROS formation was measured at 12 h after infection. (J) The effect of tempol or DPI on the hHA-mediated regulation of short poly(I·C)-induced IFN-β transcription. MDA5^−/−^ cells were transfected with the p3×Flag-EV or p3×Flag-hHB and short poly(I·C) for 12 h. Then the cells were treated with 3 mM tempol, 3 μM DPI, or PBS for 12 h. (K) The effect of tempol on RIG-I ubiquitination. HEK293T cells were cotransfected with the indicated plasmids. At 12 h after transfection, the cells were treated with 3 mM tempol for 12 h. (L and M) The effect of tempol on hHB-induced RIG-I ubiquitination. HEK293T cells were cotransfected with the indicated plasmids. At 12 h after transfection, the cells were infected with SeV and treated with 3 mM tempol for 12 h. The data represent the means ± standard deviations from three independent experiments. Significant differences are denoted as follows: *, *P* < 0.05; **, *P* < 0.01; ***, *P* < 0.001; NS, not significant (*P* > 0.05).

Next, we investigated if ROS is required for hHB-mediated regulation of the MDA5 signaling pathway. As shown in Fig. 8A and B, MDA5- or RIG-induced IFN-β transcription was significantly reduced by tempol (an ROS inhibitor). When intracellular ROS was suppressed by tempol, hHB still inhibited long-poly(I·C)-induced activation of IFN-β promoter (Fig. 8C). Accordingly, tempol treatment could not absolutely counteract the inhibition of the MDA5- or long-poly(I·C)-induced transcription of IFN-β by hHB overexpression (Fig. 8D). To exclude the unspecific effects of tempol and the disturbance of the RIG-I-mediated signaling pathway, two other ROS inhibitors, diphenyleneiodonium chloride (DPI) and *N*-acetyl-l-cysteine (NAC), were also tested in the RIG-I^−/−^ cells. After DPI or NAC treatment, hHB-mediated inhibition of the MDA5-induced or long-poly(I·C)-induced transcription of IFN-β was still observed in RIG-I^−/−^ cells (Fig. 8E). However, hHB-induced upregulation of the IFN-β promoter activation in response to short poly(I·C) was inhibited by tempol (Fig. 8F). Tempol also suppressed hHB-induced upregulation of RIG-I-induced or short-poly(I·C)-induced transcription of IFN-β (Fig. 8G). Moreover, DPI or NAC also obviously counteracted the hHB-mediated facilitation of RIG-I-induced or short-poly(I·C)-induced IFN-β transcription in the MDA5^−/−^ cells (Fig. 8H). These findings indicate that the upregulation of the RIG-I signaling by hHB is related to the hHB-induced ROS, whereas hHB can regulate MDA5 signaling through a pathway which does not entirely depend on the hHB-induced ROS.

We have previously shown that the MDA5-mediated signaling pathway is disrupted by hHB but not by hHA. However, the RIG-I- but not MDA5-mediated signaling pathway is certainly promoted by both hHB and hHA. Thus, we also verified the role of ROS in the hHA-mediated regulation of RIG-I-mediated signaling as a reference. Our results showed that hHA could also promote the intracellular accumulation of ROS (Fig. 8I). Moreover, tempol or DPI treatment counteracted the hHA-mediated enhancement of short-poly(I·C)-induced transcription of IFN-β (Fig. 8J). These results implied that the ability of hHB to promote the activation of RIG-I by inducing ROS might be a general characteristic of hemoglobin.

Furthermore, we evaluated the effects of hHB on the ubiquitination of RIG-I upon tempol treatment. Suppression of ROS by tempol significantly inhibited the total ubiquitination and the K63-linked ubiquitination of RIG-I (Fig. 8K). Consistent with previous findings, when ROS was suppressed, the ubiquitination of RIG-I was no longer enhanced by increasing hHB (Fig. 8L). To further verify the role of ROS in the hHB-mediated regulation of RIG-I activation, we investigated the effects of hHB-induced ROS on K63-linked RIG-I ubiquitination. The results showed that K63-linked ubiquitination of RIG-I was no longer enhanced by hHB when ROS accumulation was inhibited (Fig. 8M).

## DISCUSSION

Generally, RIG-I and MDA5, the cytoplasmic RNA helicase proteins, are the main sensors of RNA viruses in triggering type I IFNs in eukaryotes (3, 4). Exploring the molecular events of the RIG-I/MDA5 signaling pathway is critical for understanding complex innate immune responses against RNA viruses. In this study, we identified hHB as a novel innate immune regulator of the RIG-I/MDA5-mediated antiviral signaling pathways, which further advances our understanding of the regulatory mechanisms involved in RLR-mediated signaling pathways.

The activation of RIG-I or MDA5 is a complex regulatory process including viral RNA binding, structural rearrangement, dephosphorylation, ubiquitination, and binding to MAVS to activate downstream antiviral signaling (46–48). Despite functional overlap between the RIG-I and MDA5 pathways, our results showed that hHB could differentially regulate these signaling pathways. RIG-I and MDA5 recognize differential viral RNAs (12). In our study, hHB significantly inhibited the replication of SeV, VSV, and NDV, which are mainly sensed by RIG-I, but enhanced the growth of EMCV, which almost only activates MDA5-mediated signaling (Fig. 1 and 4). Moreover, we showed that hHB promoted RIG-I signaling and remarkably inhibited MDA5-mediated type I IFN production (Fig. 2 and 3). Therefore, it is plausible that hHB differentially regulates RIG-I and MDA5 activation upstream of the MAVS-mediated signaling. RIG-I and MDA5 share similar structural frameworks implicated in viral dsRNA recognition and detection of short and long poly(I·C) as the synthetic dsRNA analogues, respectively (35, 49). Although RIG-I recognition of viral RNA has been mostly clarified, how MDA5 recognizes viral RNA is yet to be determined. Our results demonstrated that hHB had no obvious influence on the binding of short poly(I·C) to RIG-I, but hHB acted as a direct repressor of MDA5 by interfering with the interaction between MDA5 and long poly(I·C) (Fig. 6B and C). In addition, hHB could bind to the long poly(I·C), but it failed to interact with short poly(I·C) or MDA5 and RIG-I (Fig. 6D to F). These data imply that hHB-dsRNA may compete with the MDA5-dsRNA interaction and thus negatively regulates the MDA5-mediated IFN pathway. Moreover, ubiquitination plays a critical role in the regulation of RIG-I and MDA5 activation (3). The E3 ubiquitin ligases TRIM25 and TRIM65 catalyze the K63-linked ubiquitination of RIG-I and MDA5, respectively, and thus positively regulate the RIG-I- and MDA5-mediated signaling pathways, respectively (42, 50). In the present study, we found that hHB promoted the K63-linked ubiquitination of RIG-I whereas it inhibited MDA5 ubiquitination (Fig. 6G to I). Interestingly, our results showed that hHA had a similar effect on the activation of RIG-I and that it promoted RIG-I ubiquitination after RNA virus infection. These findings suggest that the action of hHB on the MDA5-dsRNA interaction is characteristic, but the ability of hHB to facilitate the activation of RIG-I may be due to the general characteristics of hemoglobin.

Hemoglobin is the main respiratory protein in vertebrates and many invertebrates. It exerts multiple functions and plays an important role in resistance to pathogen invasion (31). In addition to functioning as a major host respiratory protein, hemoglobin can also be specifically activated by pathogens to produce ROS to constitute a part of the host defense strategy (43, 51, 52). For example, human hemoglobin significantly enhances ROS production under microbial protease stimulation but not host protease stimulation (53). ROS plays a key role in immunity and pathogen killing (54–56). The host respiratory proteins directly exploit the invasion of microbes to produce ROS, resulting in localized cytotoxicity to rapidly kill the neighboring pathogens (53, 57). Recently, the association of ROS with RLR signaling has been reported. The host cell requires ROS to efficiently trigger RIG-I-mediated IRF3 activation and IFN-β expression (58). This indicates that ROS may provide a mediator for hHB to be involved the regulation of RIG-I signaling. Here, we demonstrated that hHB increases the ROS level in cells and that the antioxidant inhibitors, including tempol, DPI, and NAC, could counteract the hHB-mediated upregulation of the RIG-I-mediated signaling pathway but could not eliminate the effects of hHB on MDA5 signaling (Fig. 8). Moreover, inhibition of ROS by tempol suppressed hHB-mediated facilitation of RIG-I ubiquitination, especially the K63-linked ubiquitination of RIG-I. These findings certify that ROS is required for hHB-mediated regulation of RIG-I ubiquitination, which indirectly promotes the activation of RIG-I signaling. Interestingly, our results showed that hHA also promotes the RIG-I-mediated signaling pathway by enhancing the intercellular ROS accumulation. These findings suggest that the action of hHB on the MDA5-dsRNA interaction is canonical but that the ability of hHB to facilitate the activation of RIG-I might be a general attribute of hemoglobin.

**FIG 9:**
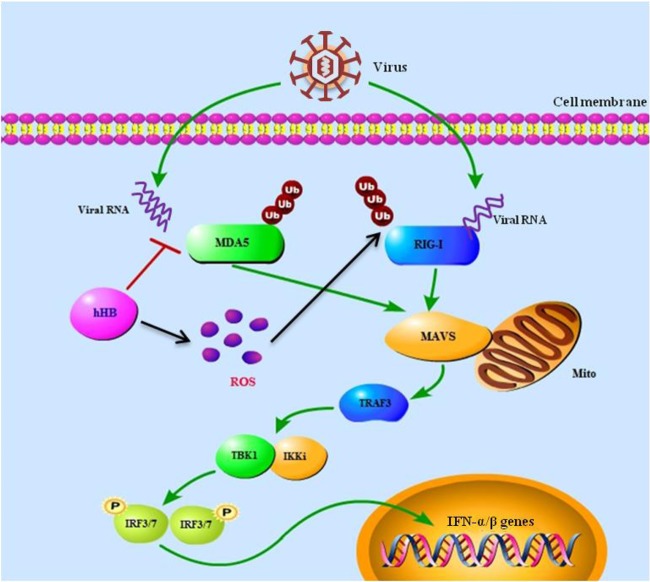
Schematic model of the hHB-mediated regulation of the RIG-I/MDA5 signaling pathways. The specific details of the model were described in the text. Ub, ubiquitin; P, phosphate group; Mito, mitochondrion.

The innate immune system has evolved various strategies to prevent harmful overproduction of type I IFNs during viral infection. Thus, several host molecules are capable of regulating type I IFN production via multiple pathways, including the RIG-I/MDA5 signaling pathway. For example, DAK is a specific repressor of MDA5-mediated signaling, and the deubiquitinating activity of A20 inhibits RIG-I-mediated signaling (19, 23). It is also worth noting that several host factors adopted multiple ways to regulate RIG-I/MDA5 signaling. For example, IFN-β levels are increased following stimulation with activators of RIG-I signaling in protein kinase R (PKR)-null cells, and the absence of PKR severely impairs MDA5-mediated IFN induction (59). In this study, we determined that hHB was a pleiotropic regulator of the RIG-I/MDA5-mediated signaling pathway. Moreover, hHB could affect RIG-I/MDA5 signaling in a direct or indirect manner. hHB directly inhibits binding of MDA5 to dsRNA and negatively regulates MDA5-mediated IFN production (Fig. 9). On the other hand, hHB is involved in the regulation of cellular oxidative stress to enhance RIG-I ubiquitination, which indirectly promotes RIG-I-mediated IFN production (Fig. 9). These findings imply that hHB contributes to protection mechanisms needed for controlling the RLR signaling pathway.

Currently, many regulators have been identified to have direct effects on a single point or a single pathway of antiviral innate immunity (60–62). For these regulators, the direct effects are appreciated and emphatically studied. However, their indirect impact on the intercellular microenvironment, such as redox state, pH, and ion leakage, are relatively ignored. In the present study, although hHB can directly inhibit type I IFN production by interfering with the MDA5-mediated signaling pathway, an hHB-induced change in the intercellular redox state will concurrently impede this inhibition by promoting the RIG-I-mediated signaling pathway. This reveals the importance of the intercellular microenvironment in the regulation of antiviral innate immunity and suggests why the effects of some regulators are always fluctuating in response to the nature of the stimuli. The indirect effects resulting from hHB’s influence on the cellular microenvironment suggest that hHB-mediated innate immune regulation may be dependent on the cellular state and stimulus types. Therefore, future work is required to further understand the regulatory mechanisms of antiviral innate immunity and to improve the effectiveness of some regulators.

In summary, we identified hHB as a novel innate immune regulator of RNA viruses through multifunctional and pleiotropic regulation of the RIG-I/MDA5 signaling pathways. On one hand, hHB promoted the RIG-I-mediated signaling pathway by enhancing RIG-I ubiquitination. On the other hand, hHB remarkably inhibited MDA5-mediated type I IFN production by interfering with the MDA5-dsRNA interaction. We mechanistically illustrated the crucial roles of hHB in regulating and protecting antiviral innate immunity. Our findings also highlight the importance of the intercellular microenvironment, such as the redox state, in the regulation of antiviral innate immunity and thus indicate that future studies are warranted to fully explore the complex induction of innate immunity.

## MATERIALS AND METHODS

### Cells, viruses, and plasmids.

HEK293T (ATCC CRL-3216) cells, a human embryonic kidney cell line, and BHK-21 (ATCC CCL-10) cells, a baby hamster Syrian kidney cell line, obtained from the American Type Culture Collection (ATCC), were cultured in Dulbecco’s modified Eagle’s medium (DMEM) with 10% fetal bovine serum (FBS). SeV and NDV expressing green fluorescent protein (NDV-GFP) were propagated in specific-pathogen-free (SPF) chicken embryos and titrated in chicken red blood cells by hemagglutination assay. EMCV and VSV-GFP were propagated in BHK-21 cells. The p3×Flag-hHB plasmid encoding the hHB protein (GenBank accession no. NM_000518.4) with three copies of the Flag tag (3×Flag) at its N terminus was constructed by cloning the hHB cDNA into the p3×Flag-CMV-10 vector (Sigma-Aldrich). The plasmids pEGFP-RIG-I and pEGFP-MDA5 encoding the RIG-I and MDA5 proteins, respectively, with an enhanced GFP (EGFP) tag at the C terminus were constructed by cloning the human RIG-I, MDA5, and MAVS cDNAs into the pEGFP-C1 vector (Clontech Laboratories). pEF-Myc-RIG-I and pEF-Myc-MDA5 encode human RIG-I and MDA5, respectively.

### Generation of hHB^-/-^, RIG-I^-/-^, and MDA5^-/-^ cells using HEK293T cells.

We generated hHB^−/−^, RIG-I^−/−^, and MDA5^−/−^ cells using a lentivirus-expressing CRISPR-Cas9 vector (lentiCRISPRv2; Addgene). The hHB-, RIG-I-, and MDA5-specific subgenomic RNA (sgRNA) sequences were the following: for hHB, 5′- GTA ACG GCA GAC TTC TCC TC-3′ (forward) and 5′-GAG GAG AAG TCT GCC GTT ACC-3′ ( reverse); for RIG-I, 5′-GGG TCT TCC GGA TAT AAT CC-3′ (forward) and 5′-GGA TTA TAT CCG GAA GAC CCC-3′ ( reverse); and for MDA5, 5′-CGA ATT CCC GAG TCC AAC CA-3′ (forward) and 5′-TGG TTG GAC TCG GGA ATT CGC-3′ ( reverse). Lenti-CRISPR virions were packaged in HEK293T cells by transfecting the psPAX2 plasmid (Addgene), the pMD2.G plasmid (Addgene), and either the lentiCRISPRv2 vector containing hHB-, RIG-I-, or MDA5-specific sgRNA or an empty lentiCRISPRv2 plasmid as a control. The suspensions were harvested at 72 h posttransfection (hpt). HEK293T cells were infected with the suspensions and treated with 1.5 μg/ml puromycin for 5 days. The cells were lysed, and hHB, RIG-I, or MDA5 expression was analyzed by Western blotting.

### Dual-luciferase reporter assay.

HEK293T cells were cotransfected with the IFN-β promoter–firefly luciferase (FLuc) reporter plasmid (pIFN-β-FLuc), p3×Flag-hHB, and the internal reference reporter TK-*Renilla* luciferase (RLuc) as an internal control (pRLuc-TK). The total amounts of the plasmid DNAs were equalized with the empty control vector p3×Flag-CMV-10 (p3×Flag-EV). At 24 hpt, the cells were infected with SeV or phosphate-buffered saline (PBS) for another 24 h. Then cells were lysed, and the activities of the reporter genes were determined using a dual-luciferase reporter assay system (10 pack; Promega). The luciferase induction mediated by IFN-β promoter (IFN-β-Luc) was presented as relative expression level of FLuc/RLuc. For the RIG-I- or MDA5-mediated response, HEK293T cells were cotransfected with pIFN-β-FLuc and pRLuc-TK as well as with pMyc-RIG-I, pMyc-MDA5, short poly(I·C) (catalog no. tlrl-picw; InvivoGen), or long poly(I·C) (catalog no. tlrl-pic; InvivoGen). The luciferase activities were measured at 24 hpt, and relative expression levels were calculated as described above.

### Real-time RT-PCR.

Total RNA was extracted from HEK293T cells using TRIzol reagent (catalog no. 15596026; Invitrogen). RNAs were converted to cDNA using Reverse Transcriptase XL (catalog no. 2621; TaKaRa). The transcription levels of IFN-β, RIG-I, and MDA5 in hHB-treated or untreated HEK293T cells with or without SeV infection were quantified by the 2^−ΔΔ^*^CT^* (where *C_T_* is threshold cycle) method (61–63). The mRNA level of glyceraldehyde-3-phosphate dehydrogenase (GAPDH) was set as an internal loading control. Primers used for the real-time reverse transcription-PCR (RT-PCR) are listed in Table 1.

**TABLE 1:** List of primers for real-time RT-PCR

Primer^*a*^	Sequence (5′–3′)
SeV N-F	AGTATGGGAGGACCACAGAATGG
SeV N-R	CCTTCACCAACACAATCCAGACC
EMCV 3D-F	TGAGCTTAGACCGATAGA
EMCV 3D-R	GATGCAAACTTTCCCAAC
GAPDH-F	GACACCCACTCCTCCACCTTT
GAPDH-R	ACCACCCTGTTGCTGTAGCC
IFN-β-F	CAAATTGCTCTCCTGTTGTGCTTC
IFN-β-R	AATGCGGCGTCCTCCTTCT
GBP1-F	TTCTTCCAGATGACCAGCAG
GBP1-R	GCTAGGGTGGTTGTCCTTGA
ISG56-F	GCCTTGCTGAAGTGTGGAGGAA
ISG56-R	ATCCAGGCGATAGGCAGAGATC
RIG-I-F	CACCTCAGTTGCTGATGAAGGC
RIG-I-R	GTCAGAAGGAAGCACTTGCTACC
MDA5-F	GCTGAAGTAGGAGTCAAAGCCC
MDA5-R	CCACTGTGGTAGCGATAAGCAG

a F: forward primer; R: reverse primer.

### ROS production assay.

Total ROS production was measured by probing with a 2′,7′-dichlorodihydrofluorescein diacetate (DCHF-DA) probe (catalog no. S0033; Beyotime Biotechnology) and was evaluated using an Enspire Multimode Plate Reader (Perkin Elmer). HEK293T cells were transfected with p3×Flag-hHB. The total amount of the plasmid DNA was equalized with the p3×Flag-EV. At 24 hpt, the cells were infected with SeV or treated with PBS for 24 h. Then the cells were washed with PBS and incubated with the DCFH-DA probes for 30 min at 37°C. After incubation, the cells were washed twice with PBS, and the 2′,7′-dichlorofluorescein (DCF) fluorescence was measured.

### Western blotting.

Equivalent amounts of each sample were resolved by SDS-PAGE and transferred to nitrocellulose membranes (Hybond-C Super; GE Healthcare). The membranes were blocked with 5% skim milk in PBS containing 0.1% Tween (PBST) and incubated for 2 h at room temperature with the primary antibodies at an appropriate dilution (anti-Flag, -Myc, -MDA5, and -RIG-I monoclonal antibodies [MAbs] at 1:1,000 and anti-hHB MAb at 1:500) (catalog no. F1804 and M4439 [Sigma-Aldrich] and catalog no. sc-134513, sc-48932, and sc-22718 [Santa Cruz]). The membranes were washed with PBST and then incubated with IRDye 800CW goat anti-mouse IgG(H+L), donkey anti-goat IgG(H+L), and goat anti-rabbit IgG(H+L) (catalog no. 926-32210, 926-32214, and 926-32211; LiCor BioSciences) or goat anti-mouse IgG-peroxidase (catalog no. A5278; Sigma) at 1:10,000 for 1 h at 37°C, and the blots were scanned using an Odyssey infrared imaging system (LiCor BioSciences) or a fluorescence/chemiluminescence imaging system (Clinx Science instruments).

### Coimmunoprecipitation (co-IP) assay.

HEK293T cells were transfected with p3×Flag-hHB together with pMyc-RIG-I or pMyc-MDA5. At 48 hpt, the cells were lysed with NP-40 buffer (catalog no. P0013F; Beyotime) with 1 mM phenylmethylsulfonyl ﬂuoride (PMSF) at 4°C for 30 min. The supernatants were collected and precleared with protein G-agarose (catalog no. 11243233001; Roche) for 2 h at 4°C. Then the mixtures were centrifuged, and the supernatants were incubated with an anti-Flag M2 affinity gel (catalog no. A2220; Sigma-Aldrich) or incubated with protein G-agarose and anti-Myc MAb or anti-RIG-I MAb overnight at 4°C. The gels were washed with NP-40 buffer, and the precipitated proteins were tested by Western blotting.

### Poly(I·C) binding assay.

HEK293T cells were transiently transfected with pMyc-MDA5 or pMyc-RIG-I and p3×Flag-hHB plasmids and lysed with NP-40 buffer containing recombinant RNase inhibitor (RRI) (catalog no. 2313A; TaKaRa). Poly(I·C) was labeled with photobiotin (catalog no. A14216; Baomanbio) using a mercury vapor lamp. The cell lysates were incubated with the labeled poly(I·C) for 4 h at 4°C. Then the biotinylated RNA-protein compounds were precipitated with Dynabeads Streptavidin (catalog no. 11205D; Invitrogen) for 1 h at room temperature using gentle rotation. After being washed with PBS, the bound proteins were analyzed by Western blotting.

### Statistical analysis.

All experiments were performed with at least three independent replicates. Results were analyzed by SPSS, version 18.0, software using Student's *t* test. A *P* value of <0.05 was considered to be statistically significant.
